# Precise *in planta* genome editing via homology‐directed repair in wheat

**DOI:** 10.1111/pbi.13984

**Published:** 2022-12-29

**Authors:** Weifeng Luo, Rintaro Suzuki, Ryozo Imai

**Affiliations:** ^1^ Genome‐Edited Crop Development Group, Institute of Agrobiological Sciences National Agriculture and Food Research Organization (NARO) Tsukuba Japan; ^2^ Faculty of Life and Environmental Sciences University of Tsukuba Tsukuba Japan

**Keywords:** genome editing, homology‐directed repair, shoot apical meristem, wheat

Genome editing (GE) via homology‐directed repair (HDR) enables genome modification with maximum flexibility. Previous gene targeting (GT) studies have demonstrated that biolistic delivery of Cas9 or Cas12a expression cassettes with donor templates into rice callus allows precise replacements or insertions at target sites using the HDR pathway (Li *et al*., 2016, 2018, 2019; Lu *et al*., 2020). Other groups also reported successful creation of GT plants in maize (Svitashev *et al*., 2016) and barley (Lawrenson *et al*., 2021). However, these strategies are only applicable to genotypes that are amenable for cell culture and regeneration. To circumvent the limitations associated with cell culture and regeneration, we recently developed the *in planta* particle bombardment (iPB) method, which permits genotype‐independent genome editing in wheat (Hamada *et al*., 2017; Liu *et al*., 2021). The iPB method utilizes shoot apical meristems (SAMs) which contain subepidermal layer (L2) cells that are destined to develop into germ cells during floral development. Successful delivery of Cas9 ribonucleoproteins (RNPs) into the SAM potentiates genome editing to occur which is heritable to the next generation (Kumagai *et al*., 2022). Since SAMs are characterized by high cell‐division activity, with many cells at the prerequisite G2/M stage for HDR, we hypothesized that HDR‐based GT is possible by delivering a designed donor DNA together with RNPs into wheat SAMs via the iPB method (Figure 1a).

**Figure 1:**
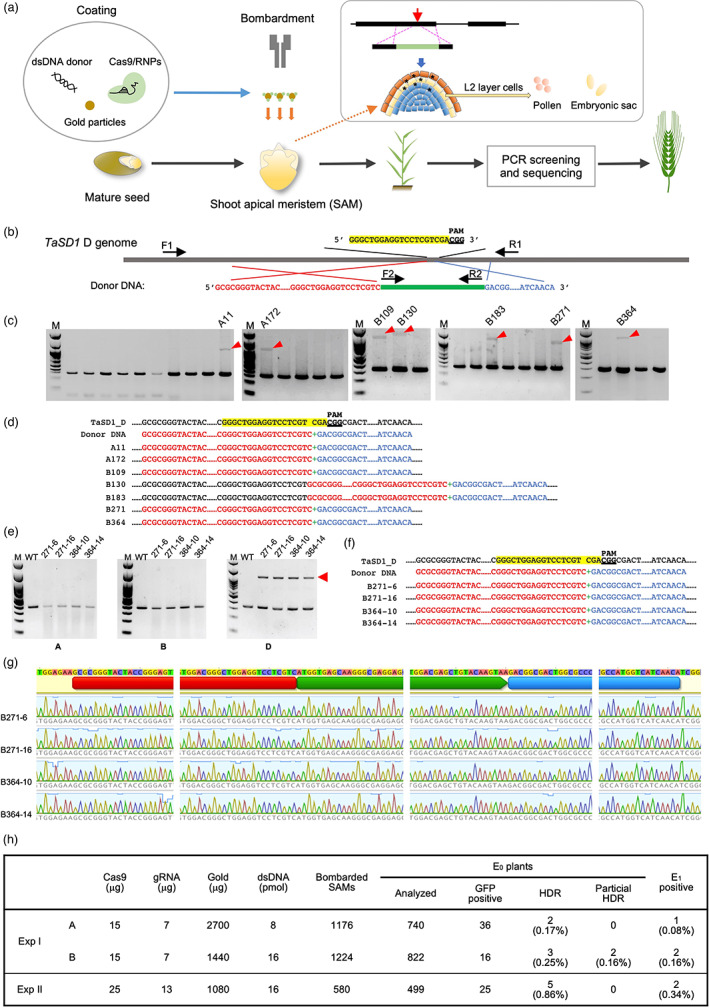
Precise target sequence replacement in wheat by *in planta* particle bombardment (iPB). (a) Precise target sequence replacement via HDR in wheat by iPB. (b) The target site of *TaSD1* and the design of the dsDNA donor. (c) PCR screening of CRISPR/Cas9‐mediated HDR plants in the E_0_ generation under condition A and B. Long PCR products designated by red arrows indicate that HDR or Non‐Homology End Joint (NHEJ) occurred at the target site in this plant. (d) The sequence alignments of mutated fragment indicated by the red arrow in (c). The left and right homology arms are marked with red and blue characters, respectively. Green ‘+’ mark denotes GFP sequence. (e) Genome‐dependent amplification of the HDR positive E_1_ plant (condition B). (f) Sequence alignments of the D genome of WT, donor DNA, and E_1_ mutants from (e). (g) Sanger sequencing results of PCR [bands designated by the red arrow in (e)]. (h) The summary of gene replacement in wheat by using the iPB method. A and B indicate coating condition A and condition B. The efficiencies are calculated based on the number of bombarded SAMs.

To explore this possibility, we designed an experiment utilizing *TaSD1* (Kumagai *et al*., 2022) as a target locus and *sGFP* as an integration gene (Appendix S1). Two arms homologous to the *TaSD1‐D* gene were linked with *GFP* and utilized as a donor (Figure 1b, Figure S1). Purified recombinant SpCas9 was mixed with chemically synthesized gRNA to form CRISPR/Cas9 RNPs. Gold particles coated with the CRISPR/Cas9 RNP and the donor DNA were then delivered into wheat embryo SAMs (cv. “Haruyokoi”) by particle bombardment (Kumagai *et al*., 2022). Two gold‐coating conditions were used: condition A, 2700 μg gold particles and 8 pmol donor DNA; condition B, 1440 μg gold particles and 16 pmol dsDNA donor. The bombarded embryos were grown *in vitro* until leaves and roots were established and materials were then subsequently grown in soil (Hamada *et al*., 2018).

The E_0_ plants grown from the bombarded embryos were subjected to screening to detect the occurrence of HDR. Genomic DNA was isolated from flag leaves of E_0_ plants and subjected to PCR screening with F2/R2 primers to detect the GFP fragment, and then F1/R1 primers were used to determine whether HDR event occurred in the plants (Figure.1b, 1c,). We totally detected seven plants that showed expected HDR products out of 2400 bombarded SAMs (Figure 1c). The amplified fragments from B130 and B183 were slightly larger than those obtained from A11, A172, B109, B271, and B364 (Figure. 1c). Sequencing of these PCR products revealed that the GFP fragment was precisely inserted into the target D genome site through HDR in plants A11, A172, B109, B271, and B364 (Figure. 1d, Figure S2). Partial HDR occurred in B130 and B183, where the right homology arm was precisely replaced and the left homology arm was inserted into the target site via non‐homologous ending joining (NHEJ) (Figure. 1d, Figure S2). Collectively, these data confirmed that the co‐delivery of RNPs and donor DNA into the SAM is a viable method to obtain HDR events in wheat.

To test whether the GT is heritable to the next generation, all E_1_ seeds from the positive E_0_ plants were harvested and subjected to genotyping. Only five positive E_1_ plants (A172‐1, B271‐6, B271‐16, B364‐10, and B364‐14) were obtained by PCR screening (Figure S3). PCR analysis with subgenome‐specific primer sets, together with Sanger sequencing, determined that plants A172‐1, B271‐6, B271‐16, B364‐10, and B364‐14 are heterozygous mutants in which the GFP fragment was precisely inserted into the target D genome site (Figure S4, Figure. 1e–g). In addition, multiple copies of randomly inserted donor DNA were identified in all five of the E_1_ plants (Table S1). With condition A (Figure 1h), we obtained two E_0_ plants with a perfect HDR event from a total of 1176 bombarded SAMs. Due to the chimeric nature of the methodology, only one of the two E_0_ plants showed inheritance of the GT to the next generation. The efficiency to obtain HDR plants was 0.17% in the E_0_ generation and 0.08% in the E_1_ generation (Figure 1h). However, with condition B (Figure 1h), a total of three perfect HDR E_0_ plants and two partial HDR E_0_ plants were obtained from 1224 bombarded SAMs, and the GT was heritable to the next generation in two of them. The HDR efficiency increased from 0.17% to 0.41% including partial HDR events in the E_0_ generation and from 0.08% to 0.16% in the E_1_ generation (Figure 1h).

In an attempt to further increase the editing efficiency, we determined that the usage of fewer gold particles (1080 μg) with a coating of more Cas9 RNPs (25 μg) resulted in higher HDR efficiencies; 0.86% in E_0_ and 0.34% in E_1_ generations, respectively (Figure 1h). These data suggested that higher amount of Cas9 RNP with smaller amount of gold particles is preferrable to increase GT efficiency.

In summary, we reported a novel approach to achieve HDR‐based genome editing by delivering Cas9 RNPs and dsDNA donor into wheat SAMs. As far as we know, this is the first report on precise HDR‐mediated GT in wheat. A total of ten E_0_ plants were obtained with perfect HDR events from 2980 bombarded SAMs. Due to the chimeric nature of the E_0_ plants, only five of them inherited the GT events to the E_1_ generation (Figure 1h). Under the best condition utilized, the efficiency for obtaining HDR plants in E_0_ and E_1_ generations reached 0.86% and 0.34%, respectively. Employing SAMs to achieve HDR circumvents the limitation of genotype dependency for optimal competency in transformation and regeneration. Here, we describe a powerful and flexible strategy that can be successfully used to introduce traits into recalcitrant commercial crops via CRISPR‐mediated HDR, without a dependency on cultivars that are highly competent in transformation and regeneration procedures. Taken together, this advancement holds great potential to expand our capabilities to rapidly improve agronomically important traits in commercial crops.

## Author contributions

W.L. and R.I., designed research; W.L. and R.S. performed research; W.L. and R.I. analysed data; and W.L. and R.I. wrote the paper.

## Conflict of interests

The authors have not declared a conflict of interest.

## Supporting information


**Figure S1** The designment of dsDNA donor.
**Figure S2** Sanger sequencing results of E_0_ plants.
**Figure S3** PCR screening for HDR plants in E_1_ generation of plant A172 (a), B271 (b) and B364 (c).
**Figure S4** Genome dependent amplification of the HDR positive E_1_ plant A172‐1.
**Table S1** The copy numbers of donor in E_1_ plants determined using qPCR.
**Table S2** Sequences of the primers used in this study.

**Appendix S1** Materials and methods.
