# Supplementary material for: Precise in planta genome editing via homology‐directed repair in wheat
Source: Plant Biotechnol J. 2022 Dec 29;21(4):668–70. doi: 10.1111/pbi.13984 (PMC10037140; doi:10.1111/pbi.13984)
Supplement: Supplementary file 1 — Figure S1 The designment of dsDNA donor. Figure S2 Sanger sequencing results of E0 plants. Figure S3 PCR screening for HDR plants in E1 generation of plant A172 (a), B271 (b) and B364 (c). Figure S4 Genome dependent amplification of the HDR positive E1 plant A172‐1. Table S1 The copy numbers of donor in E1 plants determined using qPCR. Table S2 Sequences of the primers used in this study. [file PBI-21-668-s002.pdf]

a

```
>TaSD1

 A:  AGGGTGTACCAGGAGTACTGCGGGAAGATGAAGGAGCTGTCGCTGAGGATCATGGAGCTGCTGGAGCTGAGCCAGGGCGTGGAGAAGCGCGGG
 B:  AGGGTGTACCAGGAGTACTGCGGGAAGATGAAGGAGCTGTCGCTGAGGATCATGGAGCTGCTGGAGCTGAGCCAGGGCGTGGAGAAGCGCGGG
 D:  AGGGTGTACCAGGAGTACTGCGGGAAGATGAAGGAGCTGTCGCTGAGGATCATGGAGCTGCTGGAGCTGAGCCAGGGCGTGGAGAAGCGCGGG


 A:  TACTACCGGGACTTCTTCGCGGACAGCAGCTCCATCATGCGGTGCAACTACTACCCGCCGTGCCCGGAGCCGGAGCGCACGCTGGGGACGGGC
 B:  TACTACCGGGAGTTCTTCGCGGACAGCAGCTCCATCATGCGGTGCAACTACTACCCGCCGTGCCCGGAGCCGGAGCGCACGCTGGGCACGGGC
 D:  TACTACCGGGAGTTCTTCGCGGACAGCAGCTCCATCATGCGGTGCAACTACTACCCGCCGTGCCCGGAGCCGGAGCGCACGCTGGGCACGGGC


 A:  CCGCACTGCGACCCCACGGCGCTCACCATCCTGCTGCAGGACGACGTAGGCGGGCTGGAGGTCCTCGTCGACGGCGACTGGCGGCCCGTCCGC
 B:  CCGCACTGCGACCCCACGGCGCTCACCATCCTGCTGCAGGACGACGTGGGCGGGCTGGAGGTCCTCGTCGACGGCGACTGGCGCCCCGTCCGC
 D:  CCGCACTGCGACCCCACGGCGCTCACCATCCTACTGCAGGACGACGTGGGCGGGCTGGAGGTCCTCGTCGACGGCGACTGGCGCCCCGTCCGC
                                                                                PAM

 A:  CCCGTCCCCGGCGCCATGGTCATCAACATCGGCGACACCTTCATGTAATTGCTACTGCTGCTCGTATCAG
 B:  CCCGTCCCCGGCGCCATGGTCATCAACATCGGCGACACCTTCATGGTAATTAATTGGGTCACCTTCCTTGC
 D:  CCCGTCCCCGGCGCCATGGTCATCAACATCGGCGACACCTTCATGTAATTACTCCTCTCTCAGCGTTGCT
```

b

```
TaSD1_D: GCGCGGGTACTACCGGGAGTTCTTCGCGGACAGCAGCTCCATCATGCGGTGCAACTACTACCCGCCGTGCCCGGAGCCGGAGCGCACGCT
dsDonor: GCGCGGGTACTACCGGGAGTTCTTCGCGGACAGCAGCTCCATCATGCGGTGCAACTACTACCCGCCGTGCCCGGAGCCGGAGCGCACGCT


TaSD1_D: GGGCACGGGCCCGCACTGCGACCCCACGGCGCTCACCATCCTACTGCAGGACGACGTGGGCGGGCTGGAGGTCCTCGTC GACGGCGACT
dsDonor: GGGCACGGGCCCGCACTGCGACCCCACGGCGCTCACCATCCTACTGCAGGACGACGTGGGCGGGCTGGAGGTCCTCGTC+GACGGCGACT
```

Left homology arm 169 bp

```
TaSD1_D: GGCGCCCCGTCCGCCCCGTCCCCGGCGCCATGGTCATCAACA
dsDonor: GGCGCCCCGTCCGCCCCGTCCCCGGCGCCATGGTCATCAACA
```

Right homology arm 52 bp

```
  GFP(+): ATGGTGAGCAAGGGCGAGGAGCTGTTCACCGGGGTGGTGCCCATCCTGGTCGAGCTGGACGGCGACGTAAACGGCCACAAGTTCAGCGTG
          TCCGGCGAGGGCGAGGGCGATGCCACCTACGGCAAGCTGACCCTGAAGTTCATCTGCACCACCGGCAAGCTGCCCGTGCCCTGGCCCACC
          CTCGTGACCACCTTCACCTACGGCGTGCAGTGCTTCAGCCGCTACCCCGACCACATGAAGCAGCACGACTTCTTCAAGTCCGCCATGCCC
          GAAGGCTACGTCCAGGAGCGCACCATCTTCTTCAAGGACGACGGCAACTACAAGACCCGCGCCGAGGTGAAGTTCGAGGGCGACACCCTG
          GTGAACCGCATCGAGCTGAAGGGCATCGACTTCAAGGAGGACGGCAACATCCTGGGGCACAAGCTGGAGTACAACTACAACAGCCACAAC
          GTCTATATCATGGCCGACAAGCAGAAGAACGGCATCAAGGTGAACTTCAAGATCCGCCACAACATCGAGGACGGCAGCGTGCAGCTCGCC
          GACCACTACCAGCAGAACACCCCCATCGGCGACGGCCCCGTGCTGCTGCCCGACAACCACTACCTGAGCACCCAGTCCGCCCTGAGCAAA
          GACCCCAACGAGAAGCGCGATCACATGGTCCTGCTGGAGTTCGTGACCGCCGCCGGGATCACTCACGGCATGGACGAGCTGTACAAGTAA
```

Supplementary Figure 1. The designment of dsDNA donor.
(a) The sequence alignment of the TaSD1 gene on the target site. The gRNA binding sequences characters are highlighted with yellow color. The different nucleotides in A, B and D genome are highlighted with background color. Common primer sequences are underlined. The A, B and D genome specific reverse primer sequences are highlighted with green. (b) the features and sequence of the dsDNA donor. dsDNA donor consists of 169 bp left homology arm, GFP sequence ('+' mark, 720bp) and 52 bp right homology arm. Note that the Cas9 cut site and the end of left homology arm are 1 bp difference.

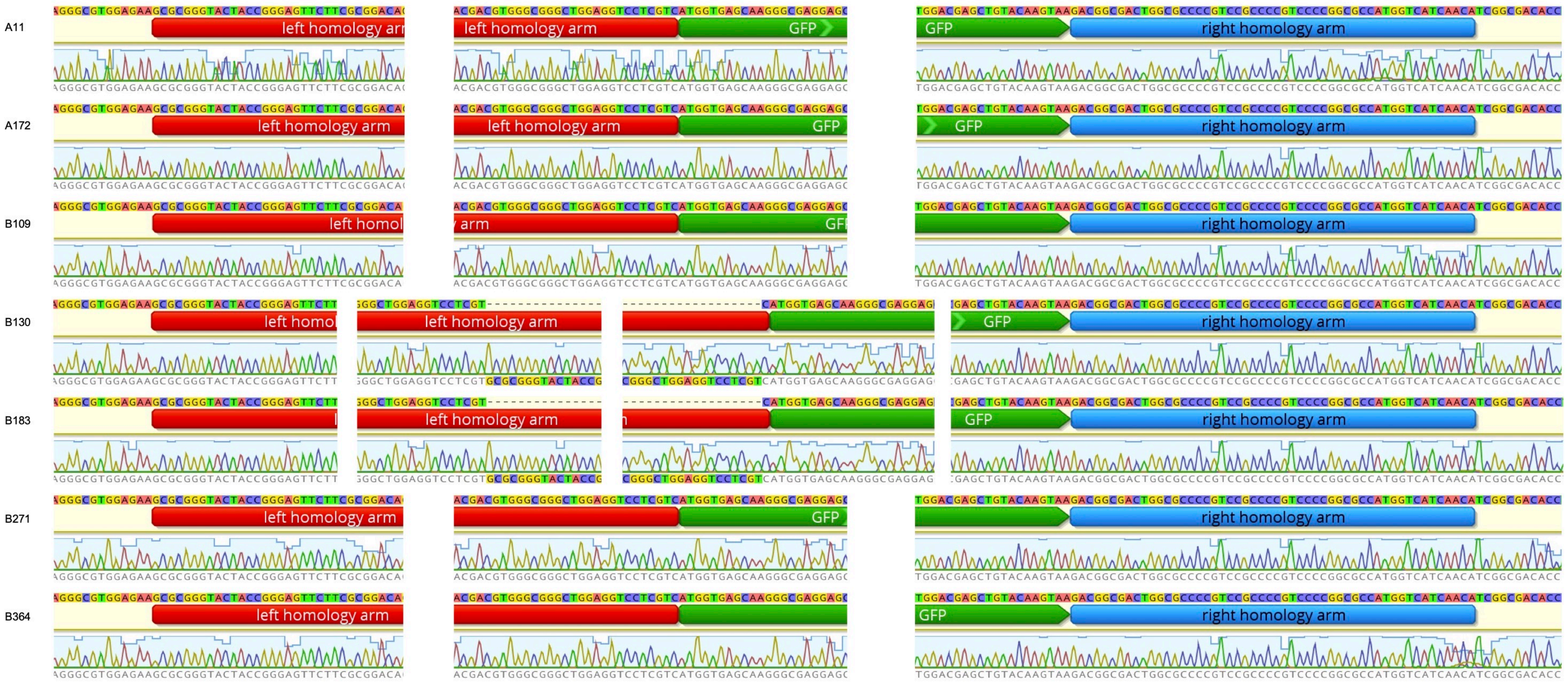

Supplementary Figure 2. Sanger sequencing results of E_0 plants.
The original Sanger sequence data of long PCR products in Figure 1(c).

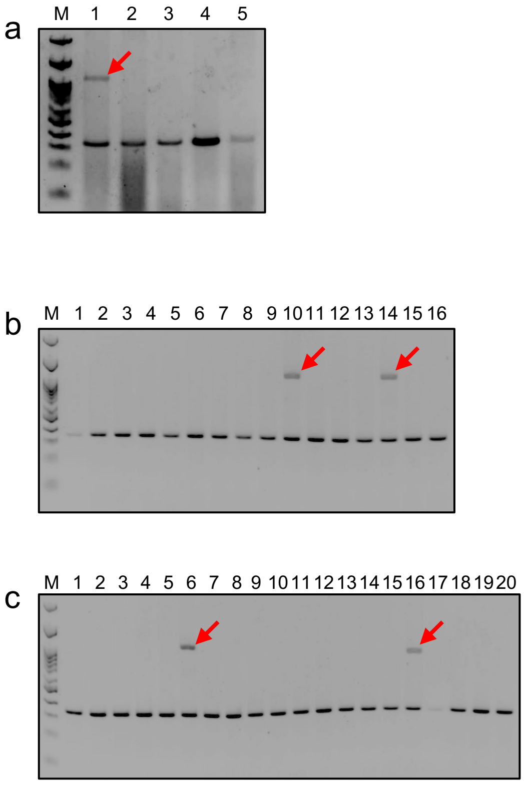

Supplementary Figure 3. PCR screening for HDR plants in E_1 generation of plant A172 (a), B271(b) and B364 (c). Totally, 5 E_1 plants of A172 (a), 16 E_1 plants of B271 (b) and 20 E_1 plants of B364 (c) were subjected to PCR screening. The PCR products are amplified by universal primer set. Red arrows indicate that HDR or NHEJ is occurred at target site in E_1 plant. A 100 bp ladder is used as a size marker.

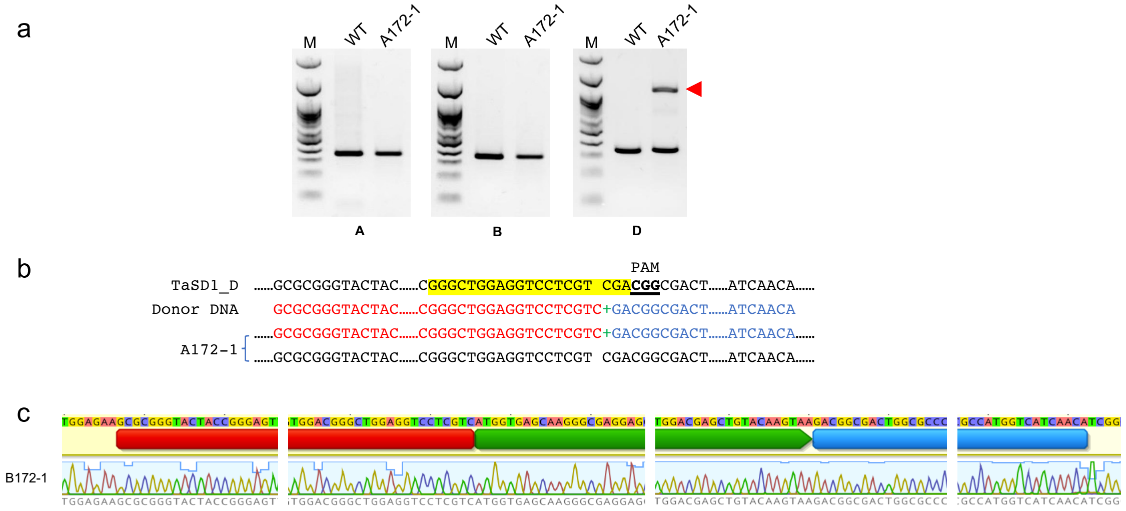

Supplementary Figure 4. Genome dependent amplification of the HDR positive E_1 plant A172-1.
(a), PCR products of positive E_1 plants are amplified by A, B and D genome specific primer sets. Red arrow indicates that the GFP gene was precisely inserted at the target site in the D genome. (b), Sequence alignments of the D genome of WT, donor DNA and E_1 mutants. (c), The original Sanger sequence data of long PCR products from (a).

Supplementarty Table S1. The copy numbers of donor in E_1 plants determined using qPCR

Mutant	Generation	Bombardment condition			Donor copy number*
		Donor	Gold particles (0.6 μm)	Cas9/gRNA	
A172-1	E_1	8 pmol	15 μL 180mg/mL	15 μg/ 7 μg	4
B271-6	E_1	16 pmol	8 μL 180mg/mL	15 μg/ 7 μg	4
B271-16					3
B361-10					3
B361-14					1

*Only random integration donors is count here.

Supplementary Table S2. Sequences of the primers used in this study.

Primer name	Primer sequence (5'-3')	Note	Purpose
SD1_F1	AGGGTGTACCAGGAGTACTG	For A, B and D genome	To amplify the TaSD1 target region.
SD1_R1	ATGAAGGTGTCGCCGATGTT		
GFP_F	ATGGTGAGCAAGGGCGAGGA	For E_0 screening	To amplify the GFP sequence.
GFP_R	TTACTTGTACAGCTCGTCCA		
donor_F	GCGCGGGTACTACCGGGAGT	For dsDNA donor application	To amplify dsDNA donor
donor_R	TGTTGATGACCATGGCGCCG		
SD1_F1	AGGGTGTACCAGGAGTACTG	For construction of dsDNA donor by overlap PCR.	To construct left homology arm
SD1_KI_Left_R	TCCTCGCCCTTGCTCACCATGACGAGGACCTCCAGCCCGC		
SD1_KI_Left	GCGGGCTGGAGGTCCTCGTCATGGTGAGCAAGGGCGAGGA		To construct GFP fragment
SD1_KI_Right	ACGGGGCGCCAGTCGCCGTCTTACTTGTACAGCTCGTCCA		
SD1_KI_Right_F	TGGACGAGCTGTACAAGTAAGACGGCGACTGGCGCCCCGT		To construct right homology arm
SD1_R1	ATGAAGGTGTCGCCGATGTT		
gRNA_F	TAATACGACTCACTATAGGGCTGGAGGTCCTCGTCGA	TaSD1	Template DNA amplification for gRNA synthesis
gRNA_R	TTCTAGCTCTAAAACTCGACGAGGACCTCCAGCC		
SD1_A_R	CTGATACGAGCAGCAGTAGC	TaSD1	A,B and D genome specific reverse primer.
SD1_B_R	GCAAGGAAGGTGACCCAATT		
SD1_D_R	AGCAACGCTGAGAGAGGAGT		
SD1_qPCR_F	CTGCTGCCCGACAACCACTA	For qPCR	To detect the donor copy number.
SD1_qPCR_R	ATGAAGGTGTCGCCGATGTT		
GFP_qPCR_F	AGAACGGCATCAAGGTGAAC		
GFP_qPCR_R	GAACTCCAGCAGGACCATGT		
