# Supplementary material for: Precise in planta genome editing via homology‐directed repair in wheat
Source: Plant Biotechnol J. 2022 Dec 29;21(4):668–70. doi: 10.1111/pbi.13984 (PMC10037140; doi:10.1111/pbi.13984)
Supplement: Supplementary file 2 — Appendix S1 Materials and methods. [file PBI-21-668-s001.pdf]

Supplementary Information for

Precise *in planta* genome editing via homology-directed repair in wheat

Weifeng Luo[1], Rintaro Suzuki[1], Ryozo Imai[1,2*].

[1]Genome-Edited Crop Development Group, Institute of Agrobiological Sciences, National Agriculture and Food Research Organization (NARO), Kannondai 3-1-3, Tsukuba, Ibaraki, 305-8604, Japan

[2]Faculty of Life and Environmental Sciences, University of Tsukuba, 1-1-1 Tennodai, Tsukuba, Ibaraki 305-8572, Japan

*Corresponding author: Ryozo Imai
e-mail: rzi@affrc.go.jp

Supplemental Materials and Methods

Preparation of SAMs.

The protocol for SAM preparation has been previously described (Hamada *et al.*, 2018).

In short, mature seeds of wheat (*Triticum aestivum* L.) 'Haruyokoi' were used in this research. To expose SAM, the coleoptile and the first three leaves were removed from imbibed seed embryos under a stereo microscope using a 34G pen needle (ϕ 0.2 mm; TERUMO, Japan). Embryos were cut off from the seeds and placed upright in a petri dish containing Murashige and Skoog (MS) basal medium. Approximately sixty embryos were placed on the center of medium for particle bombardment.

Preparation of donor DNA for HDR.

We designed a dsDNA donor fragment consisting of 169 bp left homology arm, GFP sequence (720bp) and 52 bp right homology arm targeted on the *TaSD1* gene (Figure S1). Note that the Cas9 cut site and the end of the left homology arm differ by 1 bp. The dsDNA donor fragment was constructed via overlap PCR with the designed primer sets (Table S2) and then cloned into pCR-BluntII-TOPO (Thermo Fisher Scientific, USA) and sequenced. Donor DNA was prepared from the correctly assembled plasmid template using PCR with donor_F/donor_R primer set (Table S2). The PCR reaction was conducted with KOD DNA polymerase (Toyobo), and the PCR products were subsequently purified with a DNA purification kit (TAKARA).

Preparation of Cas9 protein and sgRNA.

Recombinant *Streptococcus pyogenes* (Sp) Cas9 protein and gRNA were prepared as previously described (Kunitake *et al.*, 2019).

CRISPR/Cas9-RNP-mediated HDR using dsDNA in wheat.

The purified SpCas9 protein (15 μg) and sgRNA (7 μg) were mixed in a binding buffer (25 μL) containing 5 μL of 10×CutSmart® buffer (New England BioLabs, USA) and 1μL of RNase inhibitor (40U, Takara, Japan) and left for 10 min at room temperature. After the addition of 5 μL of *Trans*IT-LT1 transfection reagent (Mirus), the mixture was maintained for an additional 5 min at room temperature. Then, 15 μL

(condition A) or 8 μL (condition B) or 6 μL (experiment II) of 180 mg/mL gold particles (0.6 μm, Bio-Rad) were added to the RNPs mixture, tap-mixed, and kept on ice for 10 min. The gold particles were pelleted by centrifugation (3000 × g, 5s) and the supernatant was discarded. 8 pmol (condition A) or 16 pmol (condition B, experiment II) of dsDNA donor was then added into a tube and filled with Nuclease-Free water (Invitrogen, USA) to a final volume of 26 μL. The tubes were gently mixed with tapping, and the gold particles were subsequently dispersed by sonication and kept on ice for an additional 10 min. Finally, the solution was dispersed by slight sonication and 6.5 μL of the mixture was loaded onto a hydrophilic film (Scotchtint, 3M, Japan) which was quickly placed onto a microcarrier and allowed to air-dry at room temperature for 15 min.

Bombardment was conducted using a PDS-1000/He™ device (Bio-Rad, USA) with a target distance of 6.0 cm from the stopping plate. The vacuum in the chamber was 27 inches of Hg and the helium pressure was 1300 psi. Bombardment was repeated four times per plate.

Plant growth conditions.

After bombardment, the SAMs were transferred to a new MS medium and cultured for 2–3 weeks in a growth chamber under long day conditions (16 h light/8 h darkness, 22°C). The seedlings were subsequently planted in soil in pots and grown in a phytotron under long day conditions (16 h light/8 h darkness, 22 °C).

HDR-positive plant screening and sequencing analysis.

The screening strategy is shown in Figure 1b. An inner primer set (GFP_F/GFP_R) was first used to screen GFP-positive E_0 plants; and an outside primer set (TaSD1_check_F1/TaSD1_check_R1) was then used to screen HDR-positive E0 plants. Genomic DNA was extracted from the flag leaf of E_0 plant or the first leaf of the E_1 plant. PCR amplification was conducted using KOD FX Neo DNA polymerase (Toyobo, Osaka, Japan) with genomic DNA (50 ng) as template. All plants were tested individually with PCR and sequencing.

For GFP-positive plant screening, the mixture was denatured for 2 min at 98 °C in a thermocycler and then subjected to 30 cycles of amplification (98 °C for 10 s, 60 °C for 20 s, 68 °C for 40 s). For HDR-positive plant screening, the mixture was denatured

for 2 min at 98 °C in a thermocycler and then subjected to 30 cycles of amplification (98 °C for 10 s, 60 °C for 20 s, 68 °C for 60 s). The genotype of E_1 plants were confirmed by PCR using A, B, and D genome specific primer sets (Table S2).

Long PCR products were cloned into pCR-BluntII-TOPO (Thermo Fisher Scientific, USA) and sequenced on a 3130xL genetic analyzer (Applied Biosystems, USA). The sequence chromatograms were analyzed using Geneious version 10.2.6 (Biomatters). All Sanger sequencing results are provided in the Supplementary Data section.

Copy number analysis.

Genomic DNA was extracted from the E_1 plants. Since all 5 E_1 plants have heterozygous D genome (AABBDd), the mutated locus where HDR occurred were used as single copy control. GFP fragment regions were selected for copy number determination using qPCR following a documented method (Ding *et al.*, 2004).

SI References

Ding, J., Jia, J., Yang, L., Wen, H., Zhang, C., Liu, W., and Zhang, D. (2004) Validation of a Rice Specific Gene, Sucrose Phosphate Synthase, Used as the Endogenous Reference Gene for Qualitative and Real-Time Quantitative PCR Detection of Transgenes. *J. Agric. Food Chem.*, **52**, 3372–3377.

Hamada, H., Liu, Y., Nagira, Y., Miki, R., Taoka, N., and Imai, R. (2018) Biolistic-delivery-based transient CRISPR/Cas9 expression enables in planta genome editing in wheat. *Sci. Rep.*, **8**, 14422.

Kunitake, E., Tanaka, T., Ueda, H., Endo, A., Yarimizu, T., Katoh, E., and Kitamoto, H. (2019) CRISPR/Cas9-mediated gene replacement in the basidiomycetous yeast Pseudozyma antarctica. *Fungal Genet. Biol.*, **130**, 82–90.
